# High Resolution Imaging Study of Interactions between the 37 kDa/67 kDa Laminin Receptor and APP, Beta-Secretase and Gamma-Secretase in Alzheimer's Disease

**DOI:** 10.1371/journal.pone.0100373

**Published:** 2014-06-27

**Authors:** Katarina Jovanovic, Ben Loos, Bianca Da Costa Dias, Clement Penny, Stefan F. T. Weiss

**Affiliations:** 1 School of Molecular and Cell Biology, University of the Witwatersrand, Johannesburg, Republic of South Africa; 2 Department of Physiological Sciences, University of Stellenbosch, Stellenbosch, Republic of South Africa; 3 Department of Internal Medicine, University of the Witwatersrand, Johannesburg, Republic of South Africa; The Scripps Research Institute Scripps Florida, United States of America

## Abstract

Alzheimer's disease (AD) is the most prevalent form of dementia affecting the elderly. Neurodegeneration is caused by the amyloid beta (Aβ) peptide which is generated from the sequential proteolytic cleavage of the Amyloid Precursor Protein (APP) by the β– and γ- secretases. Previous reports revealed that the 37 kDa/67 kDa laminin receptor (LRP/LR) is involved in APP processing, however, the exact mechanism by which this occurs remains largely unclear. This study sought to assess whether LRP/LR interacted with APP, β- or γ-secretase. Detailed confocal microscopy revealed that LRP/LR showed a strong co-localisation with APP, β- and γ-secretase, respectively, at various sub-cellular locations. Superresolution Structured Illumination Microscopy (SR-SIM) showed that interactions were unlikely between LRP/LR and APP and β-secretase, respectively, while there was strong co-localisation between LRP/LR and γ-secretase at this 80 nm resolution. FRET was further employed to assess the possibility of protein-protein interactions and only an interaction between LRP/LR and γ-secretase was found. FLAG co-immunoprecipitation confirmed these findings as LRP/LR co-immunoprecipitated with γ-secretase, but failed to do so with APP. These findings indicate that LRP/LR exerts its influence on Aβ shedding via a direct interaction with the γ-secretase and possibly an indirect interaction with the β-secretase.

## Introduction

Alzheimer's Disease (AD) is the most prevalent neurodegenerative disorder affecting the elderly population worldwide. There are an estimated 37 million people suffering from this disease [1] and due to the lack of any effective therapies, this number continues to rise and pose more of an economic and social burden [2]. Lack of understanding of the disease causing mechanisms have resulted in great difficulties in the development of effective therapeutic interventions and as yet, the only treatment strategies are merely palliative, despite numerous ongoing clinical trials [3].

The two hallmark features of AD are the formation of extracellular amyloid beta (Aβ) plaques and intracellular neurofibrillary tangles composed of hyperphosphorylated tau protein. Oligomeric Aβ is thought to be the candidate etiological agent for AD since it has been found to mediate neurotoxicity through interactions with many other proteins [4], [5]. One such protein that has proved to be of significance in AD is the cellular prion protein (PrP^c^).

PrP^c^ is thought to have a neuroprotective role with regard to apoptosis and oxidative stress and also functions in cell signaling as well as synapse physiology [6]; however, recent reports suggest an important role for PrP^c^ in mediating the toxicity caused by the Aβ peptide in AD. Lauren *et al.* showed that PrP^c^ acts as a high affinity receptor for Aβ peptides and thus mediates the impairment of synaptic plasticity [7]. Recently reports have further verified these findings by showing that PrP^c^ was required for the neurotoxicity caused by Aβ, through impairment of long term potentiation (LTP) [8], as well as by regulating the function of the N-methyl-D-aspartate receptor (NMDAR) – a function which is hindered due to the Aβ - PrP^c^ interaction and leads to excessive activity of the receptor thereby promoting neuronal damage [9]. PrP^c^ has also been implicated in neurotoxic signalling upon interaction with Aβ whereby Fyn kinase is activated and leads to dendritic spine loss, lactate dehydrogenase activation and altered NMDAR expression on the plasma membrane of neurons [10], [11].

The cellular receptor for both PrP^c^ [12] and its infectious isoform PrP^Sc^ [13] is the 37 kDa/67 KDa Laminin Receptor (LRP/LR). This multifunctional receptor has numerous physiological roles including cell adhesion, migration, survival and proliferation (for reviews see [14], [15]). These roles are exploited by neoplastic cells whereby the receptor is involved in tumour metastasis [16], [17], [18], [19], apoptosis [20] and angiogenesis [21].

Due to its role as the receptor for PrP^c^, we examined whether LRP/LR may play some role in AD pathways. Blockage of LRP/LR with an anti-LRP/LR antibody (IgG1-iS18) or knock down of LRP/LR using anti-LRP shRNAs resulted in a significant reduction both in Aβ levels [22] and Aβ induced cytotoxicity [23]. As expression of APP, β- and γ-secretase were not affected upon antibody or shRNA treatment, an interaction between LRP/LR and one or more of the AD related proteins (APP, β- and γ-secretase) was deemed likely [22]. Since sAPPβ shedding was also impaired upon IgG1-iS18 and shRNA treatment, an interaction between LRP/LR and β-secretase was examined and co-immunoprecipitation revealed the existence of a (direct or indirect) interaction between the 2 proteins [22]. These findings revealed a novel role for LRP/LR in AD. We thus aimed to further investigate whether LRP/LR interacts with the proteins which are central to AD, namely APP, β- and γ-secretase using high resolution imaging.

## Materials and Methods

### Cell Culture and transient transfection

HEK293 cells were grown in Dulbecco's modified Eagle's medium (DMEM) (Hyclone) supplemented with 10% fetal calf serum (FCS) (Hyclone) and 1% Penicillin/Streptomycin. IMR-32 human neuroblastoma cells were cultivated in MEM (Hyclone) supplemented with 10% FCS, 1% Penicillin/Streptomycin, 1% Non-Essential Amino Acids and 2 mM L-glutamine. Cells were incubated at 37°C with 5% CO_2_. The HEK293 cell line was obtained from American Tissue Culture Collection (ATCC) while IMR-32 cells were obtained from the Fox Chase Cancer Centre. For transfections, cells were seeded onto sterile coverslips within the wells of a 6 well tissue culture dish (Corning) for all microscopy. For co-immunoprecipitation, HEK293 cells were seeded into 60 mm tissue culture dishes (Corning). Once the cells were 30–50% confluent, calcium phosphate transfection was performed as described previously [22] for HEK293 cells while *Trans*IT®-LT1 transfection reagent (Mirus) was used for the transfection of the IMR-32 cells (in accordance with the manufacturer's instructions). The procedure for the generation of the plasmids encoding LRP-dsRed (pLRP-dsRed) [24] and LRP::FLAG (pCIneo-moLRP::FLAG) [25] has been described previously. Plasmids for APP-GFP (pEGFPN1-APP770) and BACE1-GFP (pEGFPN1-BACE1) [26] were a generous gift from Dr. Bradley T Hyman, while pEGFP-PS1 (coding for PS1-GFP) [27] was a kind gift from Dr. Oskana Berezovska. For confocal microscopy, pLRP-dsRed was co-transfected with pEGFPN1-APP770, pEGFPN1-BACE1 and pEGFP-PS1 respectively in a 1∶1 ratio. pEGFP-N1 (Clonetech) and pDsRed-Express N1 (Clonetech) were used as controls. For co-immunoprecipitation, pCIneo-moLRP::FLAG, pEGFPN1-APP770 and pEGFP-PS1 were individually transfected into HEK293 cells in 60 mm tissue culture dishes and incubated for 72 hours, after which the cells were lysed for further experiments.

### Slide preparation

24 hours post transfection, coverslips containing adherent transfected cells (LRP-dsRed with APP-GFP, BACE1-GFP or PS1-GFP) were washed with PBS 3 times and then fixed with 4% Paraformaldehyde in PBS for 15 minutes. Coverslips were washed with PBS and mounted onto clean microscope slides using 75 µl Fluoromount (Sigma Aldrich). Slides were maintained at room temperature for 1–2 hours in the dark to allow the mounting medium to set.

### Confocal Microscopy

Slides were viewed using the Zeiss LSM 780 confocal microscope equipped with a GaAsp detector and images were acquired through Z-stack acquisition, with an increment of ±0.4 µm (depending on sample) between image frames. The AxioCam MRm camera was utilized to capture images. Cells expressing both LRP-dsRed and either APP-GFP, BACE1-GFP or PS1-GFP were selected and Z-stack acquisition was performed. Images were displayed as maximum intensity projections and subsequently analysed for co-localisation using 2D cytofluorograms and fluorescence intensity line profiles obtained with the use of Zen 2010 imaging software.

### SR-SIM Imaging

Slides were prepared as described and superresolution structured illumination (SR-SIM) was performed. Thin (0.1 µm) Z-stacks of high-resolution image frames were collected in 5 rotations by utilizing an alpha Plan-Apochromat 100×/1.46 oil DIC M27 ELYRA objective, using an ELYRA S.1 (Carl Zeiss Microimaging) microscope equipped with a 488 nm laser (100 mW), 561 nm laser (100 mW) and Andor EM-CCD camera (iXon DU 885). Images were reconstructed using ZEN software (black edition, 2011, version 7.04.287) based on a structured illumination algorithm [28]. Analysis was performed on reconstructed superresolution images in ZEN.

### FRET imaging

Cells were seeded onto coverslips, transfected as described above and Fluorescence Resonance Energy Transfer (FRET) acquisition and analysis was performed. Image frames were collected using confocal microscopy (LSM 780, Carl Zeiss) equipped with a LSM780 GaAsP detector, using a Plan-Apochromat 63×/1.4 Oil DIC M27 objective. Samples were excited with a 488 nm and 561 nm laser under utilization of a MBS 488/561 beam splitter. Emission was collected for the donor-GFP channel at an emission window of 495 nm–510 nm, the FRET channel at an emission window of 586 nm–600 nm and the acceptor-dsRed channel at an emission window of 586 nm–600 nm, using the lambda setting. Appropriate controls for background, donor spectral bleed-through (DSB) and Acceptor spectral bleed-through (ASB) were prepared and GFP only control images as well as dsRed only control images were acquired. Sensitized emission FRET analysis was performed utilizing FRET_plus Version 3 for ZEN 2010. FRET (N-FRET, normalized, Xia) data were generated, correcting for emission/excitation crosstalk and normalizing for the concentration of donor and acceptor. N-FRET was calculated using the Zeiss FRET plug-in for HEK293 cells, while the Image-J FRET plug-in was utilized for the IMR-32 cells, hence the slight differences in the appearance of the FRET signals in the images.

### FLAG Co-immunoprecipitation

HEK293 cells were utilized for this study as they share many similarities and features with neuronal cells [29]. Only HEK293 cells were subjected to co-immunoprecipitation due to ease of transfection. Cells transfected with pCIneo-moLRP::FLAG, pCDNA3.1APPwt or pEGFP-PS1 were lysed 72 hours post-transfection. Cell lysates from cells expressing LRP::FLAG were mixed with those expressing either APP-GFP or PS1-GFP. Lysates were subjected to a FLAG Co-immunoprecipitation assay (Sigma-Aldrich) according to the manufacturer's instructions and results were analysed via immunoblotting.

### Immunoblotting

Co-immunoprecipitation assay eluates were assessed using immunoblotting. LRP::FLAG was detected using anti-FLAG (mouse monoclonal IgG antibody) (1∶5000) (Sigma-Aldrich) and Anti-mouse HRP antibody (1∶5000) (Sigma-Aldrich). APPwt was detected using a rabbit monoclonal (Y188) antibody to Amyloid beta Precursor Protein (Abcam) (1∶3000) and PS1-GFP was detected using rabbit monoclonal (EP2000Y) antibody against Presenillin 1 (Abcam) (1∶3000). An anti-rabbit HRP antibody (1∶5000) (Sigma-Aldrich) was employed for the detection of the rabbit primary antibodies. Immunodetection was performed with SuperSignal West Pico Chemiluminescent Substrate and X-ray film (both Thermo Scientific).

## Results and Discussion

### Interactions between γ-secretase and LRP/LR

**Figure 1:**
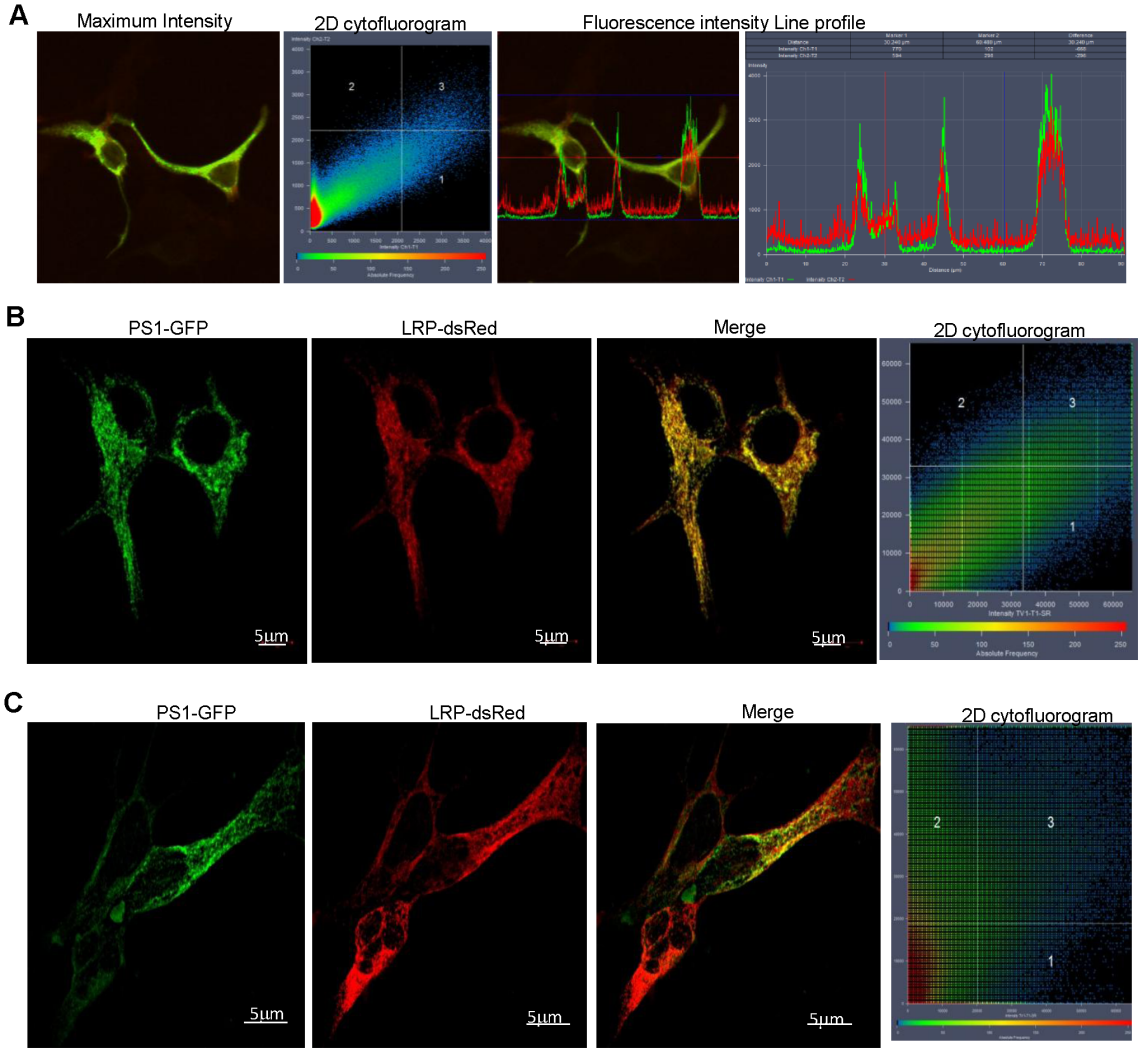
LRP/LR co-localises with PS1 of the γ-secretase complex. (A) Co-localisation between PS1-GFP and LRP-dsRed is shown for HEK293 cells in the maximum intensity profile for PS1-GFP and LRP-dsRed. The 2D cytofluorogram confirms co-localisation as a diagonal is observed passing through quadrant 3. A diagonal is indicative of a high degree of co-localisation. Line profile displaying the fluorescence intensities of the 2 colour channels along a line of interest and reveals co-localisation occurring specifically within the cytoplasmic regions. Line profile reveals aligned spectra suggestive of high degree of co-localisation. (B) SR-SIM reveals that PS1-GFP and LRP-dsRed show a high degree of co-localisation in HEK293 cells. A diagonal passes through quadrant 3 of the 2D cytofluorogram suggesting that there is still a high degree of co-localisation between the 2 proteins at this resolution (80 nm). Scale bar: 5 µm. (C) SR-SIM reveals that co-localisation between LRP-dsRed and PS1-GFP is maintained in IMR-32 cells, although the 2D cytofluorogram is skewed by the differences in transfectability with the different plasmids. Scale bars: 5 µm.

**Figure 2:**
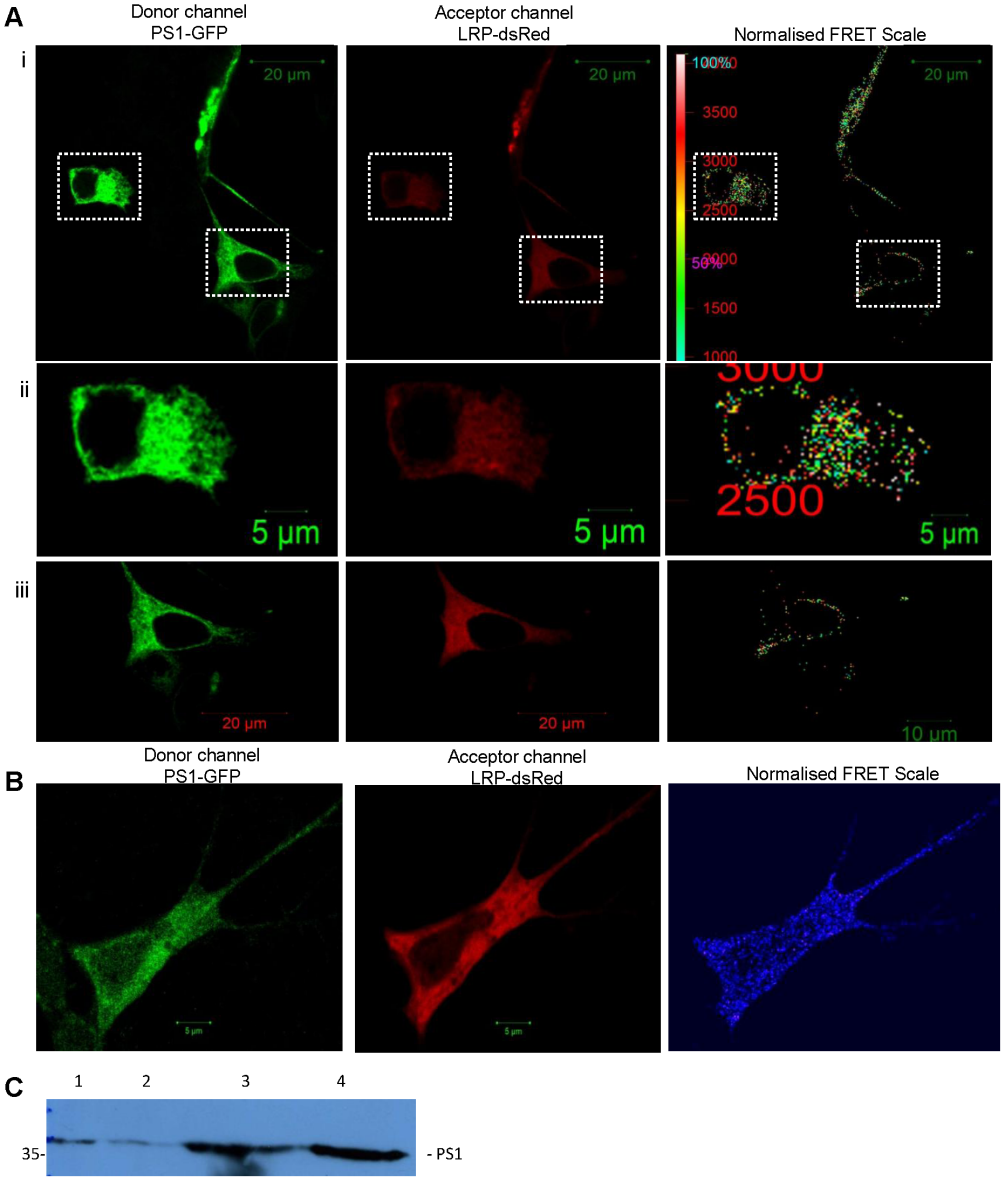
LRP/LR interacts with PS1 of the γ-secretase complex. (A)(i) FRET analysis with PS1-GFP donor and LRP-dsRed acceptor in HEK293 cells. Distinct FRET signal is detected on the normalized FRET scale. Scale bar: 20 µm. (ii) Enlarged view of single cell from (i) showing that energy is transferred in the cytoplasmic and plasma membrane regions of the cell. (iii) Enlarged view from cell in (i) showing FRET signal occurs in perinuclear membrane. (B) FRET analysis between PS1-GFP donor and LRP-dsRed acceptor performed on IMR-32 cells. Blue FRET signal is detected in the normalised FRET panel indicating an interaction is present between the two proteins. Scale bar: 5 µm. (C) FLAG co-immunoprecipitation assay was performed utilizing lysates of HEK293 cells transfected with LRP::FLAG and PS1-GFP. Immunoblot performed using rabbit monoclonal antibody (EP2000Y) for Presenillin 1. Lane (1) Non transfected cell lysates, (2) cell lysates expressing GFP alone, (3) cell lysates expressing PS1-GFP, (4) FLAG co-immunoprecipitation eluate of assay performed using cell lysates expressing LRP::FLAG and PS1-GFP. Detected bands are 36 kDa indicating PS1 dimers and not PS1-GFP.

γ-secretase is composed of 4 sub-units namely Presenilin 1 or 2 (PS1 or PS2), nicastrin, anterior pharynx defective 1 and presenilin enhancer 2 [30]. The presenilin proteins form the catalytic domain of the γ-secretase complex as they contain two aspartyl residues, within transmembrane domains 6 and 7, which are responsible for the γ-secretase cleavage of APP [31]. The 4 subunit complex is found to function predominantly within cholesterol- and sphingolipid-rich lipid raft microdomains of membranes, most notably those of the plasma membrane [32], *trans*-Golgi Network (TGN) as well as endosomes [33]. The cellular localisations of LRP/LR are limited to these same regions and the presence of this protein has also been noted in the Golgi apparatus [34]. The co-localisation observed from the maximum intensity projection between PS1-GFP and LRP-dsRed supports the notion that LRP/LR and γ-secretase appear to be located in similar cellular regions and compartments in HEK293 cells (Figure 1A). The 2D cytofluorogram accompanying the maximum intensity projection shows a clear diagonal passing through quadrant 3, indicating a high degree of co-localisation between the signal in the green and red channels which results in the observed yellow pattern of co-localisation. The line profile obtained from the maximum intensity projection of the Z-stack analysis between PS1-GFP and LRP-dsRed (Figure S1 in File S1) reveals that the spectra are well aligned and fluorescence intensity is strongest in the cytoplasm and plasma membrane. Due to the limited resolving power dictated by the wavelength utilized in laser scanning confocal microscopy, co-localisation does not necessitate functional protein – protein interactions. This can in part be overcome by an SR-SIM approach, which provides a resolution limit of approximately 80 nm (compared to the 200 nm resolution limit of laser scanning microscopy). Figure 1B reveals that even when SR-SIM is employed, a high degree of co-localisation is still maintained between these two proteins, suggesting that these proteins are indeed in close proximity to one another in HEK293 cells. The expression of both PS1-GFP and LRP-dsRed is even more distinctly defined within the cytoplasm and on the plasma membrane when observed at this resolution. Figure 1C illustrates that co-localisation between PS1-GFP and LRPdsRed is also maintained in the IMR-32 cells in similar subcellular locations as in the HEK293 cells. The lack of a clear diagonal in the 2D cytofluorogram is as a result of differing transfectability of the cells depicted in the image. As indicated, only one of the cells was successfully co-transfected with both PS1-GFP and LRPdsRed and this is where co-localisation is most evident. In order to further assess whether this co-localisation is due to protein-protein interactions, Förster Resonance Energy Transfer (FRET) was employed as in this technique, the donor and acceptor have to be within a proximity of 1–10 nm to induce FRET. A well defined FRET signal is observed in the normalized FRET scale panel (Figure 2A (i)), strongly suggesting protein-protein interaction, and not only co-localisation between γ-secretase and LRP/LR. This interaction appears to take place primarily in the cytoplasm and membrane region as revealed by the FRET signal distribution pattern, clearly observed in Figure 2A (ii), which shows a close up of the FRET signal obtained within an individual cell. LRP/LR and PS1 may be also interacting in the perinuclear region of the cell, shown in Figure 1C (iii). Furthermore, FRET was also observed in IMR-32 cells as indicated by the blue FRET signals observed in the normalised FRET panel of Figure 2B, confirming the likelihood of an interaction between PS1 and LRP. Based on these results, it is highly likely that a direct interaction does exist between LRP/LR and the PS1 subunit of the γ-secretase. In order to validate this, FLAG co-immunoprecipitation was performed using LRP::FLAG and PS1-GFP (Figure 1D) in HEK293 cells. PS1 is clearly detected in the FLAG co-immunoprecipitation assay eluate, denoting that PS1 has bound to LRP::FLAG (Figure 1D, lane 4). PS1 was detected within all of the control lanes, thus the protein visualized is certainly cellular PS1 and not PS1-GFP. The band is seen at 36 kDa which confirms that the proteins detected are most likely the homo-dimer of PS1 [35], [36], as the expected band size for PS1 is 18 kDa. This co-immunoprecipitation finding further substantiates the FRET data by confirming the interaction between LRP/LR and PS1. These findings also suggest an explanation for the altered APP processing observed upon the blockage and knockdown of LRP/LR [22], as these treatments may be interfering in or hampering the interaction between LRP/LR and γ-secretase. Further investigation is required to determine the binding sites involved in the interaction between these two proteins, in order to elucidate the role of the interaction between LRP/LR and PS1 in the context of AD pathology.

### Interactions between β-secretase and LRP/LR

**Figure 3:**
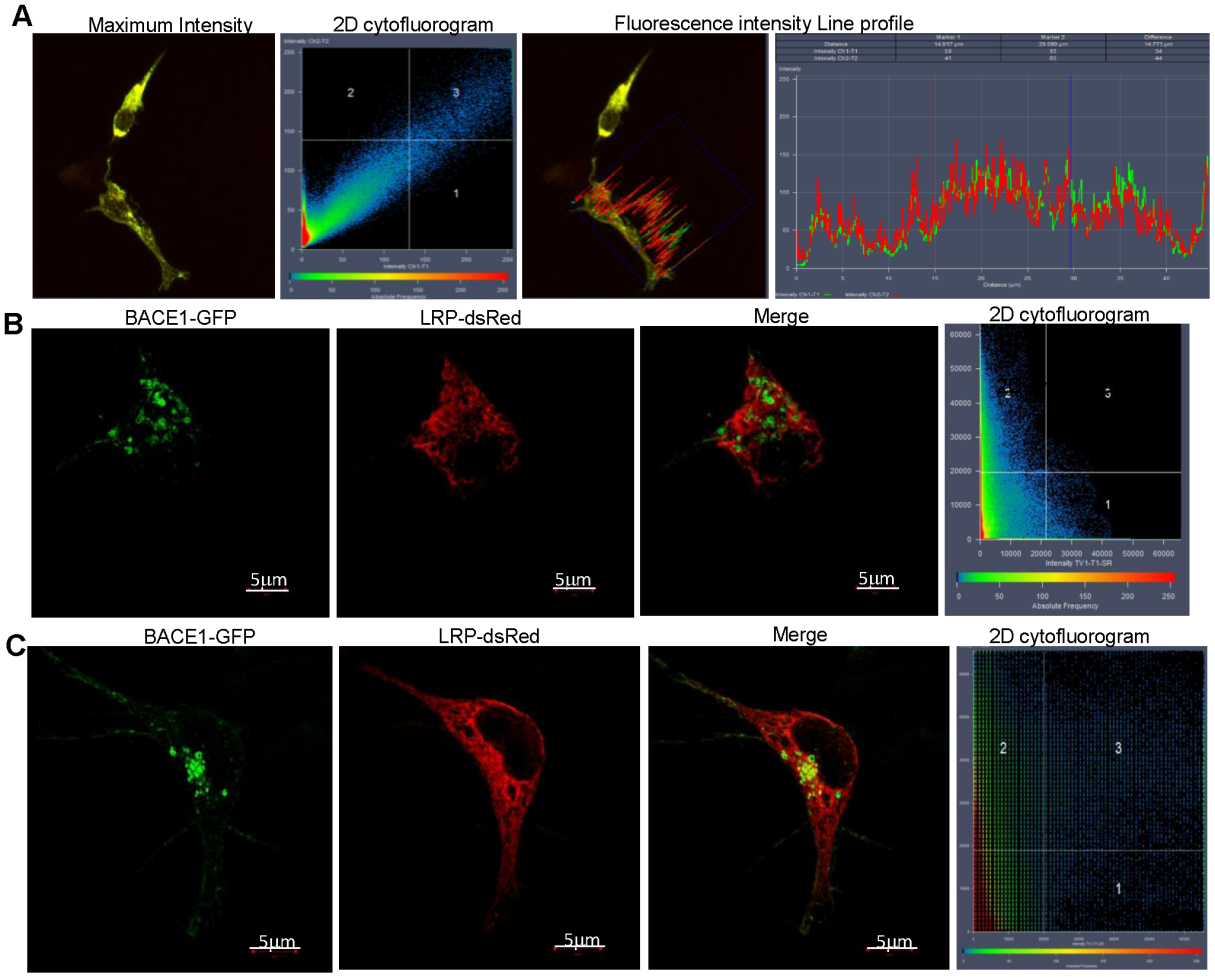
β-secretase fails to co-localise with LRP/LR at high resolutions. (A) Maximum intensity profile obtained from Z-stack analysis reveals strong cytoplasmic co-localisation between BACE1-GFP and LRP-dsRed in HEK293 cells. Diagonal in 2D cytofluorogram indicates high degree of co-localisation, as is confirmed by the line profile. (B) SR-SIM analysis of HEK293 cell expressing BACE1-GFP and LRP-dsRed. No co-localisation occurs between these proteins. The 2D cytofluorogram confirms the absence of co-localisation as there is no signal detected in quadrant 3. Scale bar: 5 µm. (C) SR-SIM employing IMR-32 cells reveals a lack of co-localisation between LRPdsRed and BACE1-GFP. Scale bars: 5 µm.

**Figure 4:**
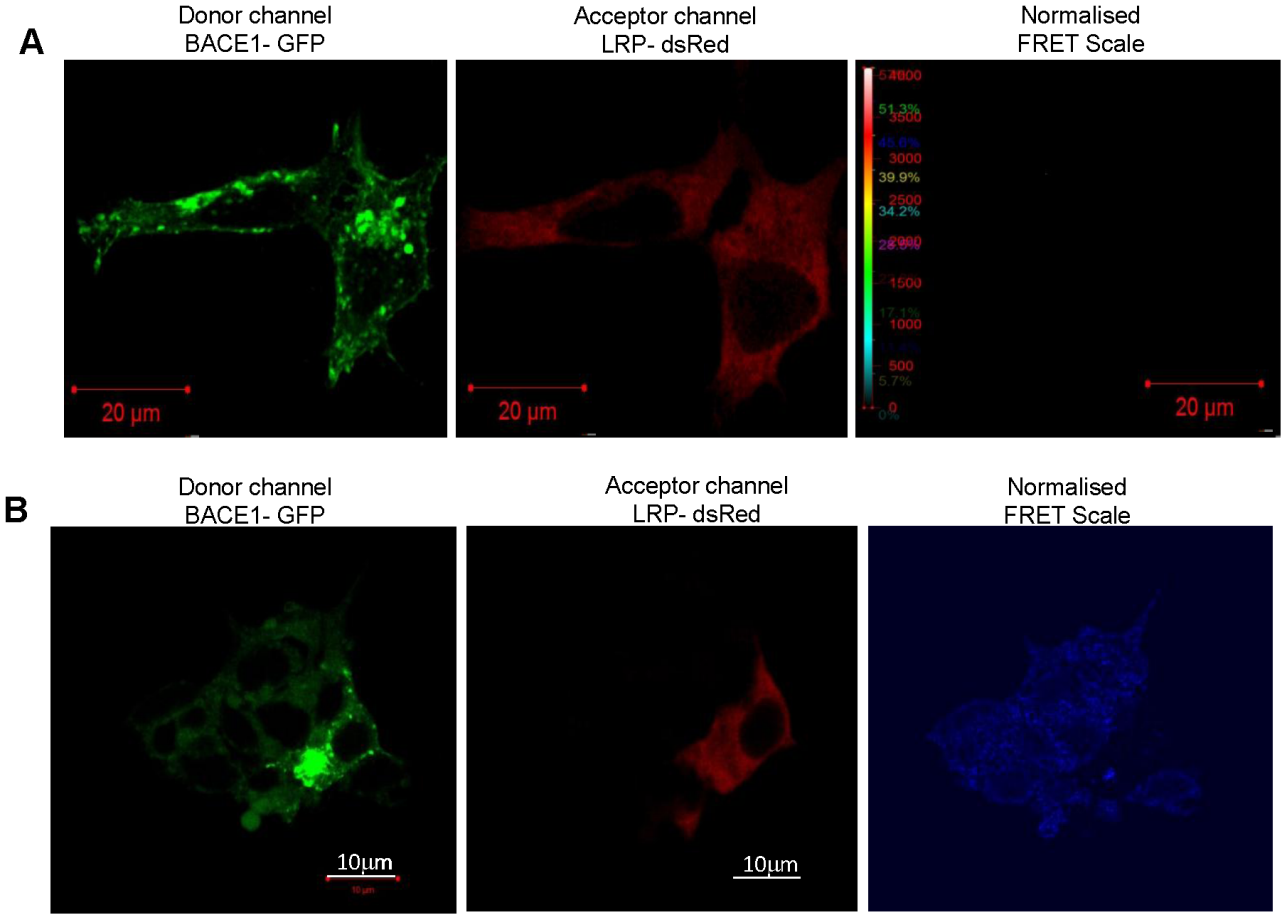
β-secretase doesn't interact with LRP/LR as revealed by FRET analysis. (A) FRET did not occur between the donor BACE1-GFP and the acceptor LRP-dsRed in HEK293 cells as no signal was detected on the normalized FRET scale. Scale bar: 20 µm. (B) Similarly, a lack of interaction was detected between LRPdsRed and BACE1-GFP when IMR-32 cells were used. Scale bars:10 µm.

β-secretase (also known as BACE1 - β-site APP cleaving enzyme) is a transmembrane aspartic protease which is predominantly localised in the Golgi and *trans*-Golgi network (TGN), as well as in endosomes, where a slightly acidic pH provides the optimal environment for APP cleavage [37]. Initial reports suggested not only co-localisation between LRP/LR and β-secretase on the cell surface, but also that these two proteins interacted, as shown by co-immunoprecipitation [22]. The findings presented in Figures 3 and 4 help to further understand the nature of the possible interactions between β-secretase and LRP/LR, where the maximum intensity projection (Figure 3A) constructed from Z-stack images (Figure S2 in File S1) shows strong co-localisation between the 2 proteins within the cellular cytoplasm – this most likely occurring in the endosomes. This is further corroborated by the line profile in which the lines representing the intensity of BACE1-GFP and LRP-dsRed are not only aligned, but also show the highest fluorescence intensities within the cytoplasm. However, when SR-SIM was employed, co-localisation was lost between LRP/LR and β-secretase, implying the proteins are situated approximately 100–200 nm from each other in both HEK293 (Figure 3B) and IMR-32 cells (Figure 3C). This diminishes the likelihood of a direct interaction between these two proteins, due to the spatial separation observed. The FRET findings further confirm this, as no FRET occurred between the BACE1-GFP donor and the LRP-dsRed acceptor within both the HEK293 and IMR-32 cells (Figure 4A and B respectively). These findings infer that the interaction that was reported between LRP/LR and β-secretase [22] is most likely an indirect interaction mediated by another cellular protein, as whole cell lysates were employed for co-immunoprecipitation studies. LRP/LR and β-secretase share several binding/interaction partners, the most notable one being PrP^c^ [38], [39]. It is possible that since LRP/LR is the cellular receptor for PrP^c^ and plays an important role in regulating endocytosis [40] and propagation [41] of PrP^c^, it may indirectly influence β-secretase activity. Another factor that may have led to the co-immunoprecipitation between LRP::FLAG and β-secretase is heparan sulphate proteoglycan (HSPG). Binding sites for HSPG have been located on LRP/LR and are implicated in regulating the interaction between LRP/LR and PrP^c^ [42]. Extensive studies have been performed on heparan sulphate, and analogues thereof, as therapeutic options for Alzheimer's Disease. Most notably this proteoglycan is seen to regulate β-secretase activity and significantly reduce the shedding of Aβ [43], [44], [45]. Thus, HSPG is another molecule which could be responsible for the indirect interaction suggested by the co-immunoprecipitation observed between LRP/LR and β-secretase [22], as it interacts with both of these proteins. PrP^c^ and HSPG could also be responsible for the decrease in sAPPβ (the cleavage product of β-secretase) levels upon anti-LRP/LR antibody and shRNA treatment [22] since they may be directly affected by these treatments, resulting in an indirect effect on β-secretase activity which they are seen to regulate.

### Interactions between APP and LRP/LR

**Figure 5:**
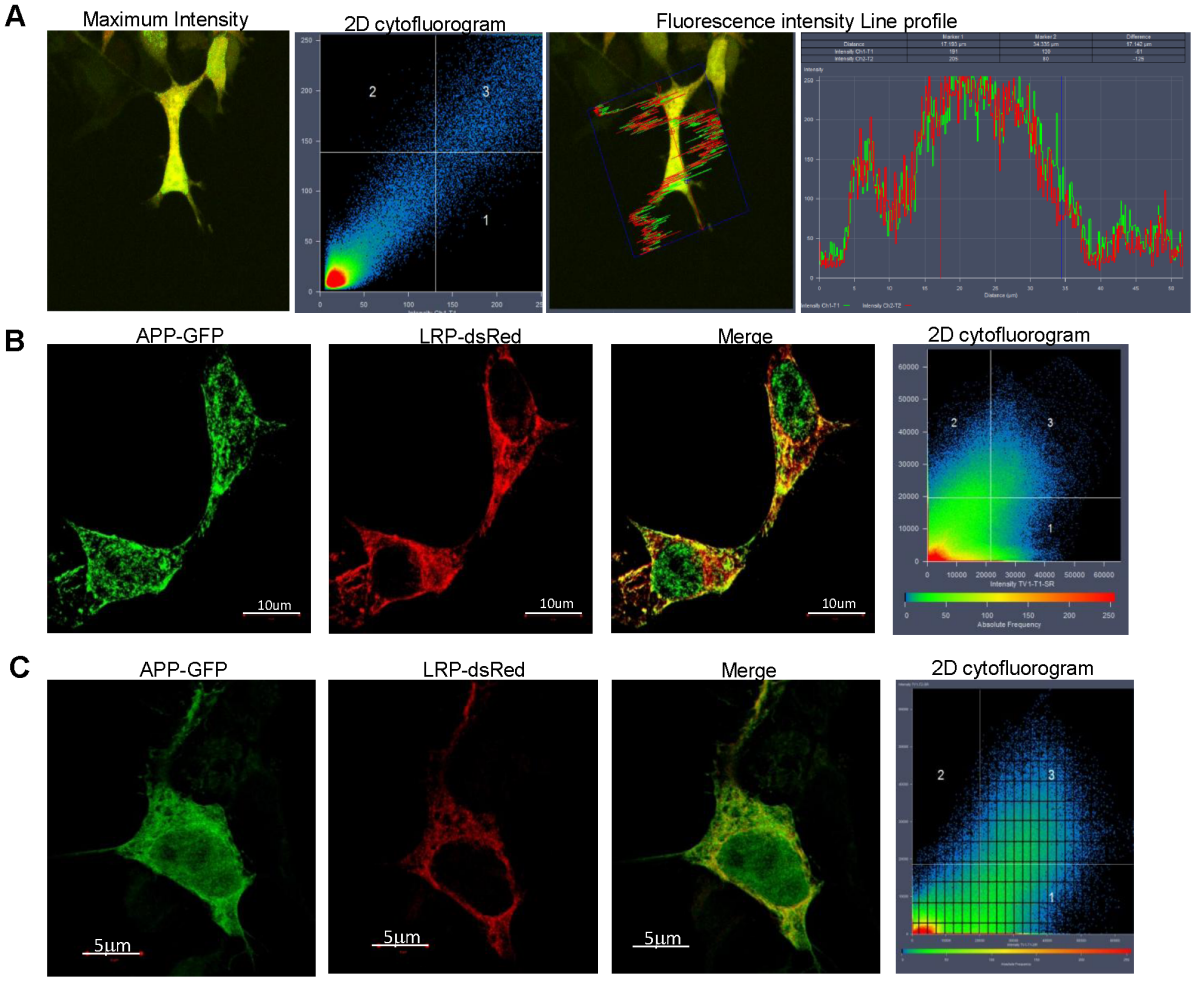
Co-localisation occurs between APP and LRP/LR. (A) Maximum intensity profile obtained from Z-stack analysis revealing co-localisation between APP-GFP and LRP-dsRed in the cytoplasm of HEK293 cells. 2D cytofluorogram reveals the co-distribution of green (APP-GFP) and red (LRP-dsRed) pixels. (B) SR-SIM analysis reveals that APP-GFP and LRP-dsRed co-localise to a lesser extent in HEK293 cells. Co-localisation is highest at the cell surface and in limited cytoplasmic locations. Scale bar: 10 µm. (C) Similar co-localisation is observed in IMR-32 cells. Scale bar: 5 µm.

**Figure 6:**
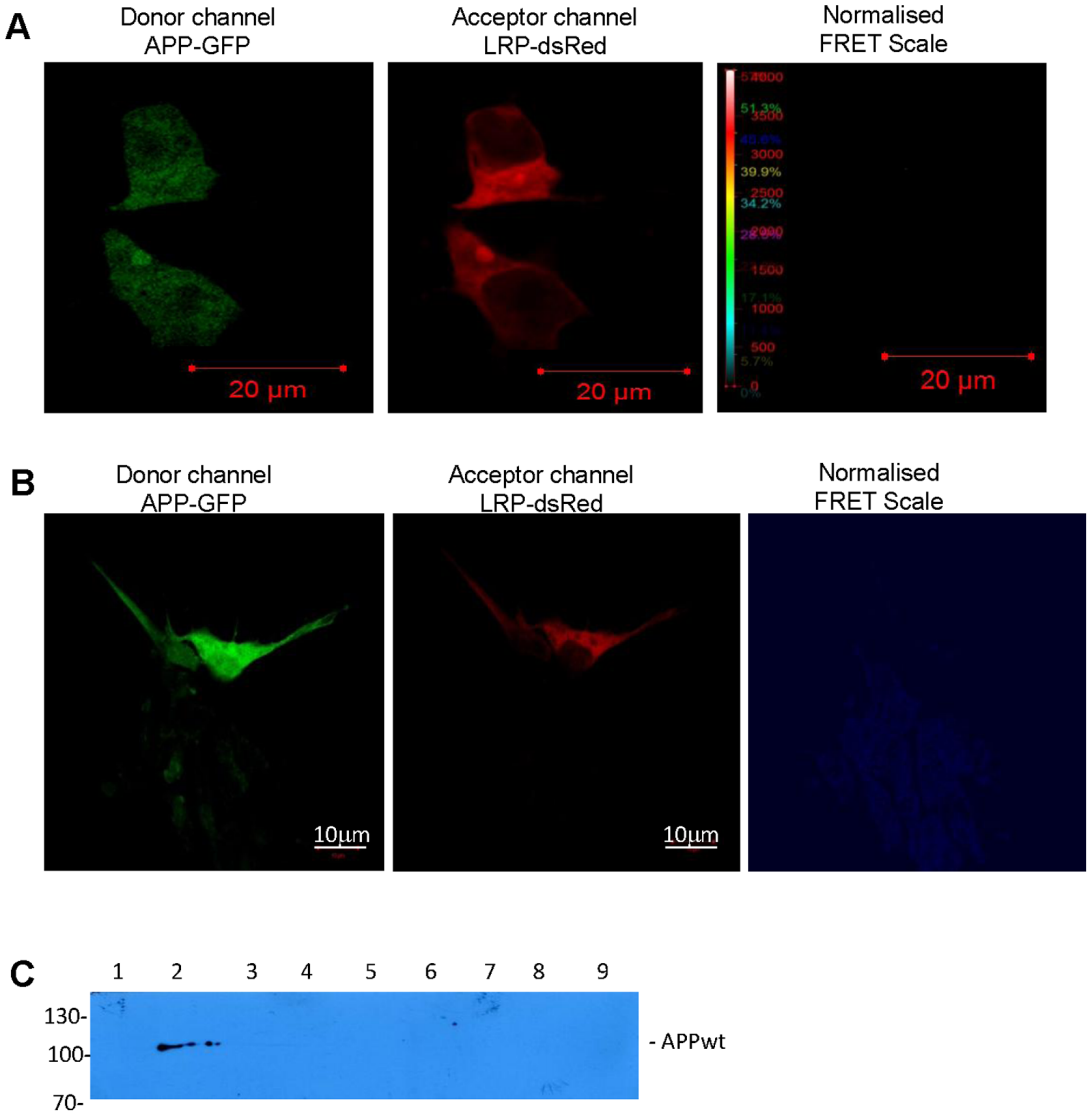
APP-GFP and LRP/LR fail to interact. FRET was performed using APP-GFP as donor and LRP-dsRed as acceptor in HEK293 cells. No FRET signal was observed on the normalized FRET scale indicating that no energy transfer occurred from BACE1-GFP to LRPdsRed. Scale bar: 20 µm. (B) Similarly, no FRET was observed between APP-GFP and LRPdsRed in IMR-32 cells. Scale bar: 10 µm. (C) FLAG co-immunoprecipitation analysis using lysates of HEK293 cells expressing LRP::FLAG and APPwt. Anti-APP antibody (Y188) was utilised for detection. Lane (1) cell lysates containing LRP::FLAG, (2) cell lysate with overexpressed APPwt (APP positive control), (3) FLAG co-immunoprecipitation assay eluate of assay performed using APPwt overexpressing cell lysates but no LRP::FLAG (i.e. Anti-FLAG M2 bead binding control), (4) BAP fusion protein (positive control for FLAG co-immunoprecipitation assay), (5) empty lane, (6) FLAG co-immunoprecipitation assay eluate of sample with LRP::FLAG and APPwt, (7–9) Wash fractions 1–3 of APPwt+LRP::FLAG FLAG co-immunoprecipitation assay post overnight binding step.

Initially, an interaction between LRP/LR and APP seemed highly likely and would provide an explanation for the effects seen on Aβ shedding upon antibody and shRNA treatment of LRP/LR [22]. Initial findings also suggested that these two proteins co-localise when detected on non-permeabilised cells (indicating cell surface co-localisation) and within the cells (indicating intracellular co-localisation) [22]. Z-stacking was performed to further verify the sub-cellular localisations of these proposed interactions and to assess whether they were maintained throughout the cell (Figure S3 in File S1). Maximum intensity projections were constructed from the images obtained via Z-stacking and revealed a degree of overlap between the LRP-dsRed with APP-GFP signal as indicated by the resulting yellow colour when the images are merged (Figure 5A). The line profile constructed from the maximum intensity projection of the Z-stack between APP-GFP and LRP-dsRed, shows the intensity of the red and green fluorescence along the line passing through the HEK293 cell of interest. These findings suggest a complete spatial overlap between the fluorescence observed in both the green and red channels. The observed intensity of both fluorescent channels is highest in the cytoplasm which further confirms the initial assumption that LRP/LR and APP are located in similar cellular locations. Findings in literature further support these data as it is known that APP is transported to the plasma membrane, where it is subsequently endocytosed from lipid raft regions into early endosomes [46], [47]. Moreover, LRP/LR is known to also be located in the lipid raft [48] as well as in early endosomes [40]. Our SR-SIM data indicates a lower degree of co-localisation between APP and LRP both in HEK293 (Figure 5B) and IMR-32 cells (Figure 5C), which appears to be confined to the plasma membrane and certain cytoplasmic regions, presumably the endosomes in which both APP and LRP/LR are localised. This suggests the proteins are indeed in close proximity to one another, resulting in the distinct co-localisation pattern. FRET was employed to further assess the viability of an interaction between LRP/LR and APP. This analysis revealed that no FRET occurred between APP-GFP and LRP-dsRed, since signal detection was lacking on the normalized FRET scale for both HEK293 (Figure 6A) and IMR-32 cells (Figure 6B). These findings indicate that no interaction takes place between APP and LRP/LR at this 1–10 nm scale. FLAG Co-immunoprecipitation using a FLAG tagged LRP/LR variant (LRP::FLAG) further confirmed these results as APP was not detected in the eluate of the assay (Figure 6C, lane 6). The results obtained from figure 3 collectively suggest that although LRP/LR and APP are found in similar cellular locations in which they co-localise, the proximity between them is still present when SR-SIM is employed (i.e. 80 nm), but lost at the nanometer scale required for FRET to take place, thus indicating that it is highly unlikely for any interaction to occur between these two proteins.

### Interactions between GFP and LRP/LR

**Figure 7:**
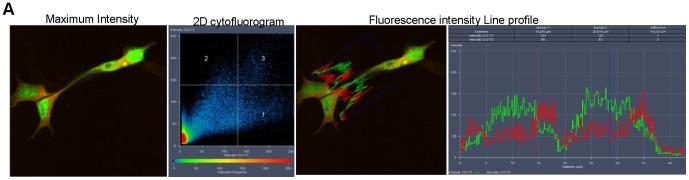
LRP/LR fails to co-localise with GFP. (A) Maximum intensity projection obtained from Z-stack analysis of GFP and LRP-dsRed shows poor co-localisation between the 2 proteins. This is further confirmed by the lack of a clear diagonal passing through quadrant 3 and the line profile which shows no alignment between the 2 colour channels.

GFP was used a negative control for these studies as it failed to co-localise with LRP/LR. This is evidenced by the maximum intensity projection (Figure 7A) obtained from Z-stack analysis (Figure S4 in File S1). The 2D cytofluorogram reveals a complete absence of a diagonal passing through quadrant 3 confirming the lack of co-localisation between GFP and LRP-dsRed. The line profile obtained shows two distinct patterns of fluorescence lacking any co-alignment of the red and green channels further confirming the low degree of co-localisation between these proteins. These findings also validate the co-localisation seen between LRP and APP, β- and γ-secretase, as the observed co-localisation is as a result of the proximity of LRP/LR to the AD proteins and not simply due to the GFP labels.

## Conclusions

In light of this study, we have found that LRP/LR is seen to co-localise with APP, β- and γ-secretase both on the cell surface and intracellularly within the cytoplasm. This co-localisation is limited to 200 nm when observed for BACE1 and LRP/LR, to 80 nm between APP and LRP/LR and less than 10 nm for LRP/LR and PS1. These results reveal a novel interaction between LRP/LR and the PS1 catalytic subunit of the γ-secretase complex (as confirmed by co-immunoprecipitation) and suggest that the previously observed interaction between LRP/LR and BACE1 is likely an indirect interaction only. These findings cumulatively highlight the role of LRP/LR in Alzheimer's Disease.

## Supporting Information

File S1 **Contains Figures S1–S4.** Figure S1, Z-stack analysis of co-localisation between PS1-GFP and LRP-dsRed. Figure S2, co-localisation between BACE1-GFP and LRP-dsRed as ascertained by z-stack analysis. Figure S3, Z-stack analysis of APP-GFP and its co-localisation with LRP-dsRed. Figure S4, lack of co-localisation between GFP and LRP-dsRed as evidenced by z-stack analysis.
(DOCX)
