# Supplementary material for: High Resolution Imaging Study of Interactions between the 37 kDa/67 kDa Laminin Receptor and APP, Beta-Secretase and Gamma-Secretase in Alzheimer's Disease
Source: PLoS One. 2014 Jun 27;9(6):e100373. doi: 10.1371/journal.pone.0100373 (PMC4074076; doi:10.1371/journal.pone.0100373)
Supplement: File S1 — Contains Figures S1–S4. Figure S1, Z-stack analysis of co-localisation between PS1-GFP and LRP-dsRed. Figure S2, co-localisation between BACE1-GFP and LRP-dsRed as ascertained by z-stack analysis. Figure S3, Z-stack analysis of APP-GFP and its co-localisation with LRP-dsRed. Figure S4, lack of co-localisation between GFP and LRP-dsRed as evidenced by z-stack analysis. (DOCX) [file pone.0100373.s001.docx]

**
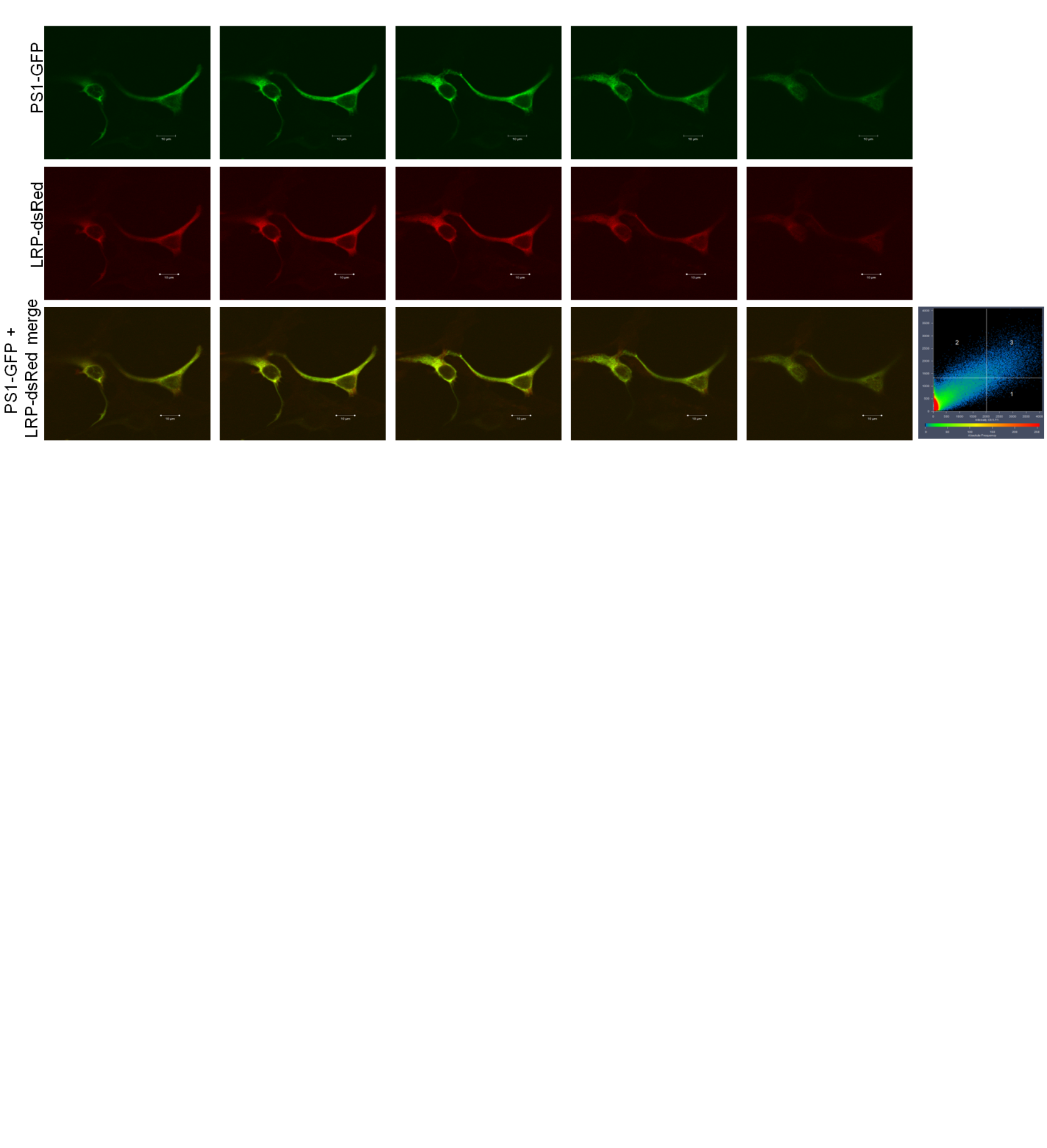
**

**Figure S1. LRP-dsRed and PS1-GFP co-localise in the cytoplasmic and cell membrane surface of HEK293 cells.** Z-stack analysis of images captured using HEK293 cells expressing PS1-GFP (top row), LRP-dsRed (middle row) and merge of both proteins (bottom row). The merge and 2D cytofluorogram both reveal a high degree of co-localisation between LRP-dsRed and PS1-GFP particularly within the cytoplasmic and plasma membrane region of the cells. A clear diagonal passes through quadrant 3 of the 2D cytofluorogram confirms the co-localization. Sections: 1.1μm; scale bars: 10μm.


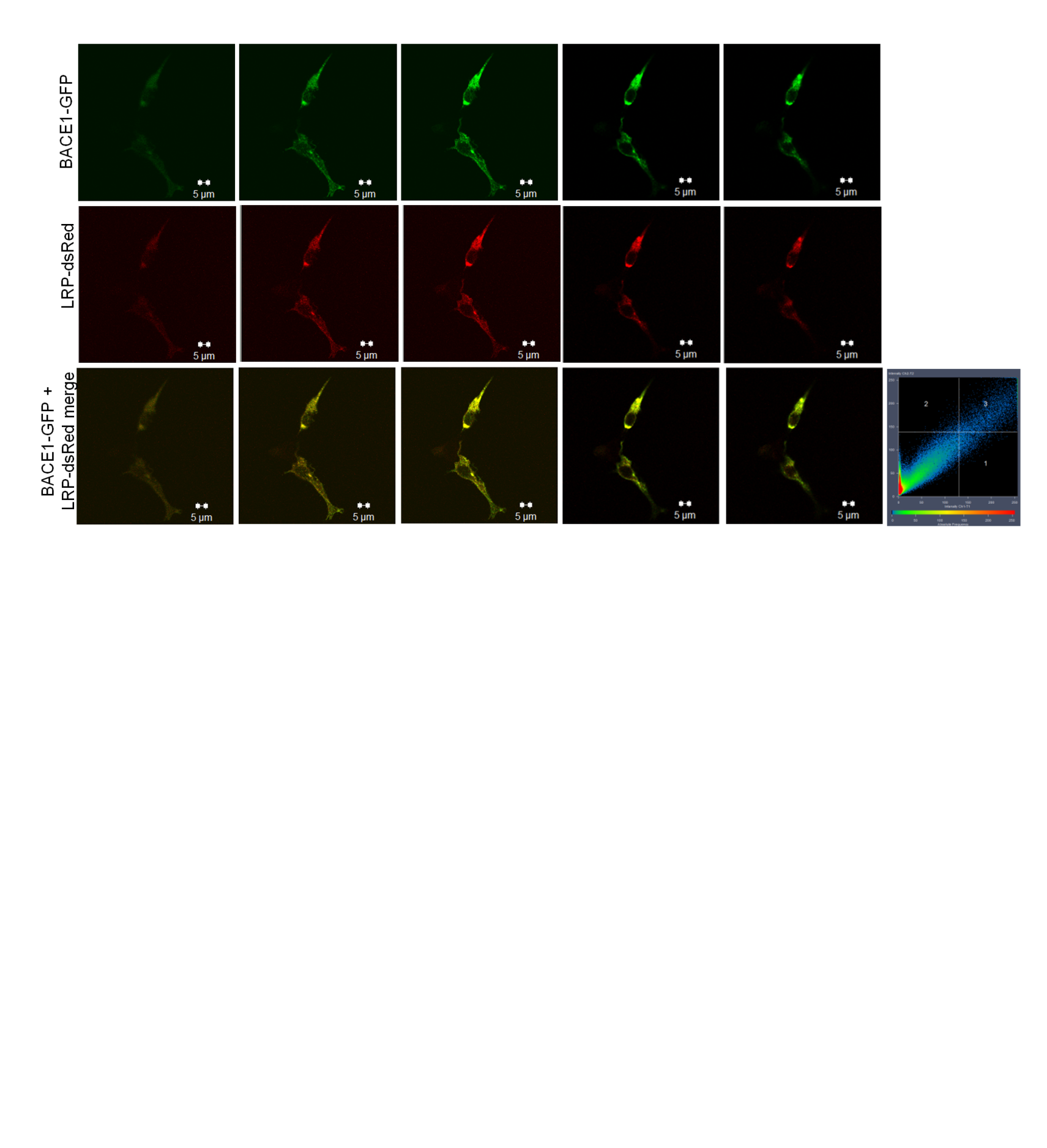


**Figure S2. LRP-dsRed and BACE1-GFP co-localise in the cytoplasm of HEK293 cells.** z-stack analysis revealing signal distribution of BACE1-GFP (top row), LRP-dsRed (centre row) and the merge of both proteins (bottom row). Distinct co-localisation exists between LRP-dsRed and BACE1-GFP within cytoplasmic regions of HEK293 cells. 2D cytofluorogram reveals a weak diagonal through quadrant 3 suggesting that co-localisation does occur but to a lesser degree. Sections: 0.39μm; scale bars: 5μm.


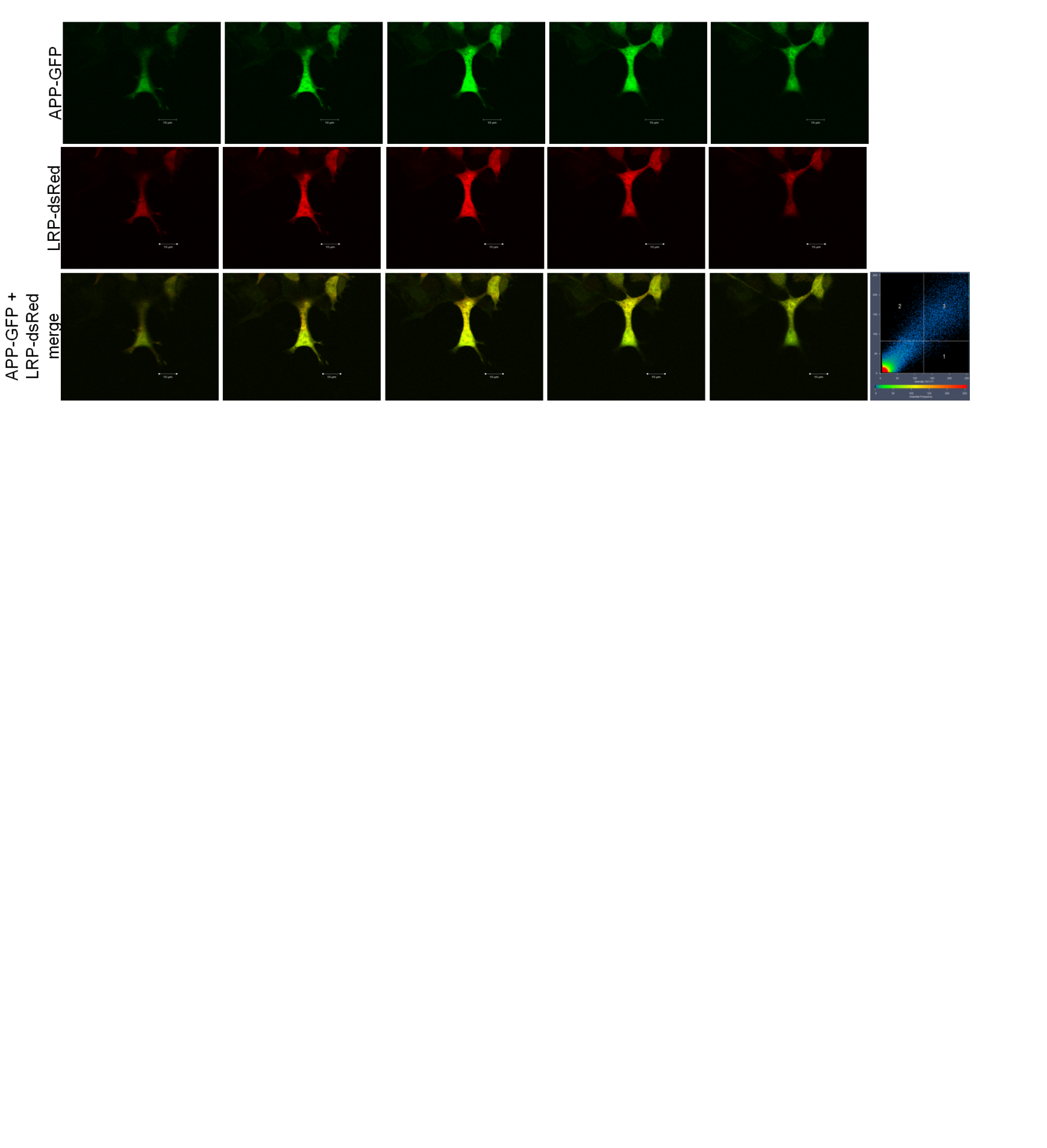
**Figure S3. LRP-dsRed and APP-GFP co-localise in the cytoplasm of HEK293 cells.** Z-stack image analysis representing subcellular localisations of APP-GFP (top row) and LRP-dsRed (middle row). Merged image (bottom row) shows APP-GFP and LRP-dsRed co-localising in the cytoplasmic regions. 2D-cytofluorogram confirms this result as is indicated by a diagonal passing through quadrant 3. Sections: 1μm; scale bar: 10μm.


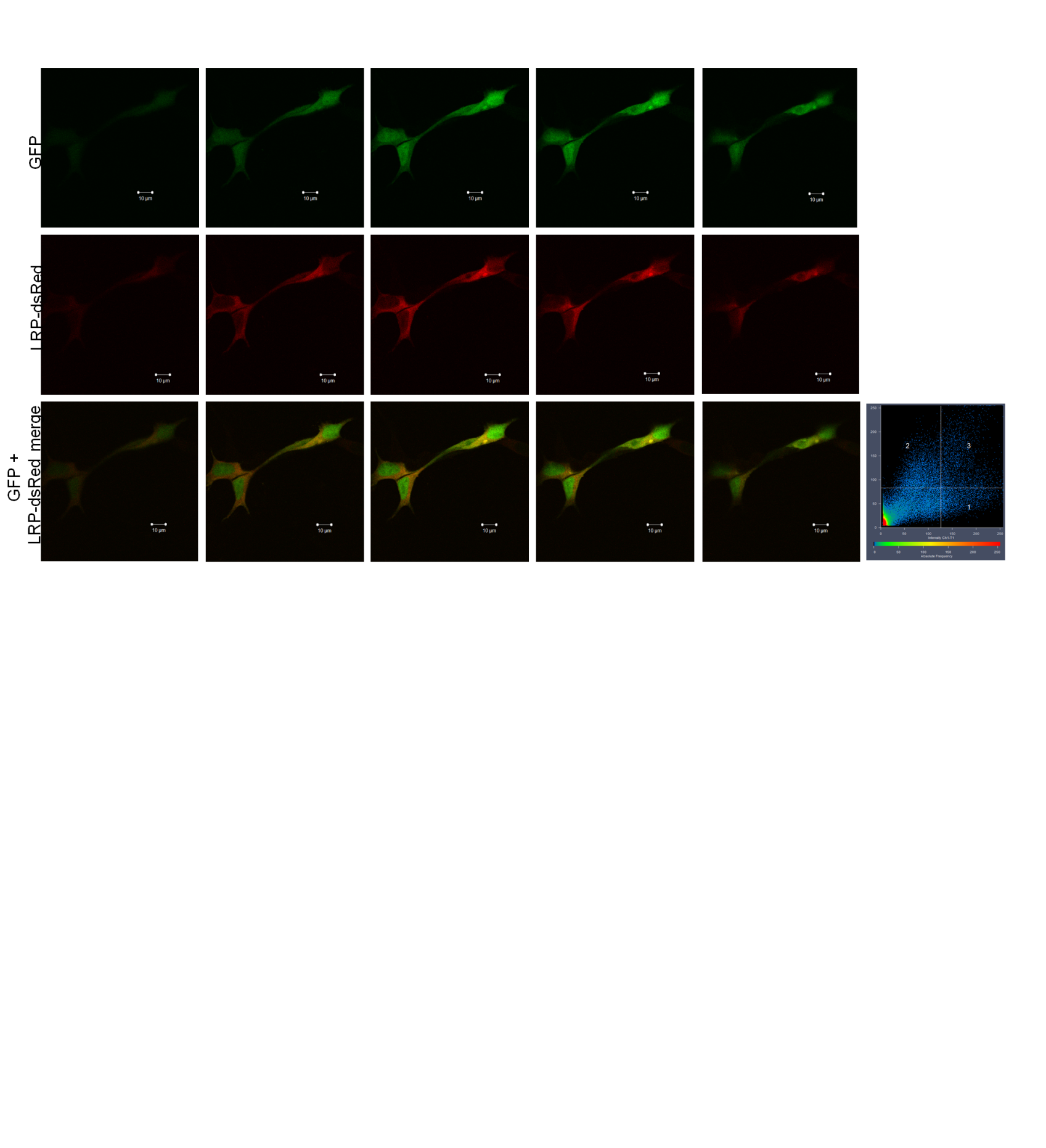


**Figure S4. LRP-dsRed and GFP fails to co-localise.** HEK293 cells were transfected with either pEGFPN1, pLRP-dsRed or both plasmids. Z-stack analysis was then performed to analyse whether LRP-dsRed co-localized with only GFP. Images show that the 2 proteins fail to co-localise as there is a weak yellow signal in the merge images. The 2D- cytofluorogram shows weak co-localisation as most signals occur in quadrants 1 and 2 (representing the green and red fluorescent channels). Very little signal is detected in quadrant 3 (the quadrant representing the yellow from the overlap of green and red fluorescence). Sections: 1μm; scale bars: 10μm.
